# Supplementary material for: Phylogenize2: robust phylogenetic methods link genes to phenotypes across host-associated and environmental microbiomes
Source: bioRxiv. 2026 Jul 16:2026.07.15.738685. Preprint. [Version 1] doi: 10.64898/2026.07.15.738685 (PMC13404670; doi:10.64898/2026.07.15.738685)
Supplement: 1 [file NIHPP2026.07.15.738685v1-supplement-1.pdf]

bioRxiv preprint doi: https://doi.org/10.64898/2026.07.15.738685; this version posted July 16, 2026. The copyright holder for this preprint (which was not certified by peer review) is the author/funder, who has granted bioRxiv a license to display the preprint in perpetuity. It is made available under a CC-BY 4.0 International license.

Supplemental Material

Supplemental Figure 1: Snakemake workflow graph detailing the steps in Phylogenize2 database preparation, showing the “marine” MGnify database as an example.

Supplemental Figure 2: Phylogenetic comparison of the MIDASv1 database with three MGnify collections across archaea (phylogenetic tree from GlobDB).

Supplemental Figure 3: Runtime and number of MMSeqs2 matches between GlobDB protein clusters and the UniRef50, FESNov, and UHGP databases, showing a plateau in sensitivity.

Supplemental Figure 4: Violin plots showing the distribution of Sourmash scores for members of the same species, genus, and family.

Supplemental Figure 5: Full length multiple sequence alignment of the polar marine Flavobacteriaceae hit MGYG000448108_02082 against experimentally validated sequences from the same protein family.

Supplemental File 1: Multiple sequence alignment file of the experimentally validated protein family sequences for the closest BLAST match and Phylogenize2 hits in the mouse high-fat diet study.

Supplemental File 2: Multiple sequence alignment file of the experimentally validated protein family sequences for the polar mesopelagic *Flavobacteriaceae*’s Phylogenize2 hits, and closest BLAST match.

Supplemental Table 1: Positive gene associations with murine gut *Muribaculaceae* on high-fat diet that were significant ($q<0.05$) via Phylogenize2, the uncorrected linear model, and/or POMS.

Supplemental Table 2: Positive gene associations with polar mesopelagic *Flavobacteriaceae* that were significant ($q<0.05$) via Phylogenize2, the uncorrected linear model, and/or POMS.

Supplemental Table 3: BLAST results containing sequence similarity between Phylogenize2 hits and the experimentally validated proteins from the xanthine/CO/aldehyde oxidoreductase family.

Supplemental Table 4: All pre-made databases currently available for Phylogenize2.

Supplemental Table 5: Sourmash thresholds used for Supplemental Figure 4.

Supplemental Table 6: Faith's phylogenetic diversity (PD) of top mouse Muribaculaceae hits.

Supplemental Table 7: Faith's phylogenetic diversity (PD) of top marine Flavobacteriaceae hits.

Supplemental Table 8: Genome neighborhood analysis for thioredoxin proteins identified as significant on a high-fat diet.
